# Potential of bacteriocins produced by probiotic bacteria isolated from tiger shrimp and prawns as antibacterial to *Vibrio*, *Pseudomonas*, and *Aeromonas* species on fish

**DOI:** 10.12688/f1000research.13958.1

**Published:** 2018-03-29

**Authors:** Feli Feliatra, Zainal Abidin Muchlisin, Hiwan Yuda Teruna, Widya Rahmi Utamy, Nursyirwani Nursyirwani, Andi Dahliaty

**Affiliations:** 1 Marine Microbiology Laboratory, Department of Marine Science, Fisheries and Marine Sciences Faculty, University of Riau, Riau, Indonesia; 2 Department of Aquaculture, Faculty of Marine and Fisheries, Syiah Kuala University, Banda Aceh, Indonesia; 3 Deptartment of Chemistry, Faculty of Mathematics and Natural Sciences, University of Riau, Riau, Indonesia

**Keywords:** potency, bacteriocin, probiotic, tiger shrimp, prawn, antibacterial, pathogen

## Abstract

**Backgrounds**: Bacteriocin has been used widely in industry as a biopreservative agent. The objective of the present study was to investigate the potency of Bacteriocin isolated from tiger prawn *Penaeus monodon* and freshwater shrimp *Macrobrachium rosenbergii* as an anti-bacterial on fish.

**Methods**: A total of ten candidates of probiotic bacteria consisted of five isolates from tiger shrimps (H1, H2, H3, H4, H5) and five isolates from freshwater prawns (W1, W2, W3, W4, W5) were evaluated. Bacteriocin wasBacteriocin was produced by centrifugation at a speed of 150 rpm and at 37 °C for 24 hours. The bacteriocin extract was purified by adding sulphate ammonium salt {(NH4) 2SO4} at 80% of the saturation level. Bacteriocin activity was determined using a diffusion method against pathogenic bacteria *Vibrio alginolyticus*, *Aeromonas hydrophillaAeromonas hydrophilla* and *Pseudomonas stutzeri*. Bacteriocins were analyzed usinganalyzedusing High Performance Liquid Chromatography (HPLC) and Fourier Transform Infra-Red (FTIR). The data were subjected to analysis of variance (ANOVA) and followed with Duncans multiple range test.

**Results**: Bacteriocins produced by bacteria isolate H4 from tiger prawn indicated the highest bacteriocin activity againstbacteriocin activity against *Pseudomonas stutzeri *at *stutzeri *at the diameter of inhibition zone of 887.10 ± 409.24 mm ^2^/mL. While isolate W2 from freshwater shrimp indicated inhibition zone of 1466.96 ± 127.62 mm ^2^/mL. Both bacteriocins were purified by chromatography column using Sephadex LH-20.

**Conclusion** **s**: This finding showed that bacterial isolates H4 and W2 have the potential to produce bacteriocins which inhibit the pathogenic bacteria. FTIR analysis showed an amide group at wave number 1652cm ^-1^ contained in the bacteriocins of isolates H4 and W2.

## Introduction

Probiotics are beneficial microbes for living organisms and, in certain quantities, have a positive impact on health ^1, 2^. Probiotic bacteria play a vital role in suppressing the growth of pathogenic microbial populations. Probiotic bacteria including lactic acid bacteria have an ability to produce several antimicrobial compounds such as lactic acid, diacetyl, hydrogen peroxide, carbon dioxide and bacteriocins ^3, 4^.

Bacteriocins are antimicrobial peptides which act as antibacterial compounds against bacterial pathogens ^5^. Bacteriocins can inhibit Gram positive and Gram negative bacteria such as *Salmonella* species (sp.), *Escherichia coli*, *Vibrio* sp., *Shigella* sp., *Aeromonas* sp. and *Pseudomonas* sp ^6^. Bacteriocins have an important role against microbes as a bactericidal as they are resistant to heat and maintain activity in an acidic environment, and low temperature during storage does not affect bacteriocin activity ^5^. Bacteriocins can be damaged by degradation from proteolytic enzymes ^7^.

Lately the use of bacteriocins has become of interest due to their potential use as a preservative in the food industry, especially in fermented foods. Nisin, for example, is one of the bacteriocins produced by lactic acid bacteria of *Lactococcuslactis* that has been known and recognized as safe to use, and can degrade pathogenic and spoilage microbes. Therefore, it could improve the quality and shelf life of food products ^8^. This study examines extracts of bacteriocins produced by probiotic bacteria isolated from tiger shrimp and prawn which have an ability to inhibit pathogenic bacteria. The findings in this study will help researchers to inform fish farmers in preparing food for fish and shrimp populations in order to prevent pathogens, improve food efficiency and to increases the animals’ immune system.

## Methods

### Sample collection

A total of ten isolates of probiotic bacteria were collected, consisting of five isolates from tiger shrimp and five isolates from prawn ^9^, which were from the collection of the Marine Microbiology Laboratory of the Faculty of Fisheries, University of Riau, Indonesia. ID numbers for each isolates are (GenBank): H1: KY995544, H2: KY995545, H3: KY995546, H4: KY995547, H5: KY995548, W1: KY995549, W2: KY995550, W3: KY995551, W4: KY995552 and W5: KY995553. This study was conducted from April-September 2016. Pathogenic gram-negative bacteria, *Vibrio alginolyticus* was ontained from Brackish water Research Institute in Jepara, Indonesia, while *Aeromonas hydrophila* and *Pseudomonas stutzeri* were purchased from Quarantine Centre in Pekanbaru, Indonesia.

The Nutrient Agar (NA; Merck, Kenilworth, NJ, USA, Cat.No. 1.05450.0500), Nutrient Broth (NB; Thermo Scientific Oxoid, Waltham, MA, USA, Cat.No.CM0001), Thiosulfate Citrate Bile Sucrose (TCBS; Merck, Cat.No.1.10263.0500), Trypticase Soy Agar, (TSA;Merck, Cat.No. 1.05458.0500), Trypticase Soy Broth (TSB, Merck, Cat.No. 1.05459.0500) were used in this study.

Glycerol, glasswool, paper disc 6mm (Macharey-Nagel, Düren, Germany, MN827ATD), gel filtration chromatography using Sephadex LH-20 (Sigma-Aldrich, St-Louis, MO, USA - Offer sigma-GE17-0090-01), methanol and other chemicals were used in accordance with the laboratory procedures.

### Refreshment of probiotic bacteria

Probiotic bacteria can grow in acidic conditions so each process was performed in physiological acidic pH solutions. In this study, probiotic bacteria were grown at pH 4. The probiotic bacteria was cultured in the following manner: a tube filled with the 500μL physiological solution of pH 4 was inoculated with probiotic bacteria aseptically at concentration of 10 ^8^ cfu/mL. The mixture was homogenized and left to stand for 5 minutes. One loop full of the mixture was streaked on NA media. Media containing the bacteria were incubated at 37 °C for ± 24 hours.

### Production of bacteriocins

Liquid medium, nutrient broth of 300 mL volume was used for the production of bacteriocins ^10^. The Medium was sterilized in an autoclave at a pressure of 15 psi, at 121°C for 15 minutes. After one day at room temperature, the medium was inoculated with 5% of probiotic bacteria that had previously been incubated overnight at the optical density (OD) at 600nm ~ 0.1 (v/v) which was equivalent to 10 ^7^ CFU/mL (OD measured with Thermo Scientific GENESYS 10S Uv-Visible) ^11^. The inoculated media was then fermented in a shaking incubator at a speed of 150 rpm for ± 24 hours at 37 °C. After incubation, the fermented medium was cooled in a refrigerator at 5–10°C for ± one hour. The crude bacteriocin extract from the medium by centrifugation (Hitachi, Tokyo, Japan – CS150FNX) at 10,000 rpm for 10 min at 4°C. The supernatant was then separated by filtration through glasswool. The cell-free supernatant (extracted bacteriocins) produced, was tested for activity against pathogenic bacteria by using a disc diffusion method. Some of the supernatants was precipitated by the addition of 80% salt ammonium sulfate [(NH4) 2SO4], and then was tested for activity in the same manner, and finally was analyzed by using High-performance liquid chromatography (HPLC).

### Purification of bacteriocins

Bacteriocin was precipitated from the crude extract by the addition of 80% salt ammonium sulfate [(NH4) 2SO4] in a cold condition (temperatures of 5 °C to 10 °C) while stirring gently to achieve 80% saturation and was then left overnight. The precipitate was then separated from the filtrate by centrifugation (Hitachi – CS150FNX) in a cold state at 13,000 rpm for 10 minutes. After centrifugation, the precipitate was added with 0.05 M phosphate buffer solution at pH 7.0, and it was ready to be used for the bacteriocins activity test. The rest was put in microtubes and was stored in a freezer (-20 °C).

### Bacteriocin activity test

Bacteriocin activity was tested by the diffusion agar method by using a paper disc of 6 mm pore size against pathogenic bacteria *V. alginolyticus*, *A. halophyla,* and *P. stutzeri*. One mL of each of pathogen inoculum (OD 600nm ~ 0.1, which is equivalent to 10 ^7^ CFU/mL ^11^ was inoculated into a test tube containing 15 mL of NA medium and was then vortexed. The inoculated medium was poured into a Petri dish and was allowed to solidify. A total of 50 mL of bacteriocins (bacteriocin extract both before and after precipitation) was dropped on paper discs and was allowed to dry. Amoxsan® 30μg from local pharmacy in Pekanbaru Indonesia was used as positive control and sterile liquid medium as the negative control. The dried paper discs were placed on pathogenic-inoculated NA media. After incubation for ± 24 hours, the bacteriocin activities were indicated by clear zone formed around the discs. The diameter of the clear zones was measured by using calipers. Inhibitory activity against pathogenic bacteria of extracellular fluid was calculated as AU (Activity Unit). One AU/mL was the area of inhibition zone per unit volume of bacteriocinsamples tested (mm ^2^/ml). Bacteriocin activity can be calculated using the following equation ^5^:

Bacteriocin Activity (mm ^2^/mL) = $\frac{\mathrm{Lz}–\mathrm{Lc}}{V}$

Where:
Lz = diameter of clear zone area (mm ^2^/ml)
Lc = Disc diameter (mm ^2^/ml)
V = Sample Volume (mL)

### Gel filtration chromatography

Gel filtration or gel permeation is a protein separation technique based on molecular size ^12^. This technique used a column measuring 2.5 × 50 cm (Sephadex LH-20) as steady phase and methanol as mobile phase. A total of 9 g Sephadex LH-20 was weighed and then was added to 50 mL of methanol. The mixture was stirred gently until dissolved. The dissolved Sephadex was poured into the column until the marked limit. The eluent was collected in a beaker containing a mixture of Sephadex, it was then poured again into the column until Sephadex expanded and solidified for 20 minutes. Afterward, the bacteriocin was inserted into the column through the column wall carefully and waited until the sample penetrated through pores of the gel Sephadex LH-20 pores. The eluate was then collected in a marked vial as the first fraction, and the next eluent was collected in vials containing 20 drops until final-clear eluent reached. Small molecules will enter the pores of the gel Sephadex and move slowly, while the large molecules will move faster because it cannot enter and retaining the gel. Thus, the large molecules will emerge as the initial component. Products of each of separated-bacteriocin fractions were finally analyzed by using FTIR spectroscopy.

### HPLC (high-performance liquid chromatography) analysis

Bacteriocins produced after precipitation in ammonium sulphate was then analyzed using HPLC. A spectrophotometer ultraviolet (UV) detector (Thermo Scientific Genesys 10S) was used for the analysis of bacteriocins at a wavelength of 210 nm and 250 nm. Wavelength selection was based on preliminary measurement using UV spectrophotometer in The Research Laboratory of Enzymes, Fermentation, and Biomolecular, following the research performed by Masuda *et al*., ^13^. The analysis used Shimadzu HPLC system of UFLC Shim Pack C18 series with column size of 4.6 mm × 250 mm (Shimadzu, Kyoto, Japan).

### Statistical analysis

The data were subjected to one-way analysis of Variance (ANOVA) ^14^ followed by Duncan multiple range testat significance levels of 95%. The analysis was performed using a SPSS ver.20 software.

## Results

### Bacteriocins activities

Inhibitory activity of bacteriocins was expressed as the inhibition zone per unit volume of samples tested (mm ^2^/mL). Table 1 shows the activity of bacteriocins extract of tiger shrimp against pathogenic bacteria *V. alginolyticus, A. hydrophila* and *P. stutzeri*. Statistical analysis indicated that the activity was not significantly different (P> 0.05), the highest activity was indicated by bacteria isolate H1 against *V. alginolyticus*. However, the activity was not significantly different (P> 0.05) from isolates H2 and H5, which was 674.65 mm ^2^/mL, but it was significantly different (P> 0.05) from isolates H3 and H4 ( Table 1). The highest activity of bacteriocin crude extract against *A. hydrophila* was found at 319.91 mm ^2^/mL from isolate H4, which was not significantly different (P> 0.05) from isolates H2, H3, and H5. However, it was significantly different (P> 0.05) from isolate H1 (P <0.05). The highest inhibitory activity against *P. stutzeri* was indicated by isolate H5 which was significantly different (P <0.05) from four other bacteria.

**Table 1.** Summary of crude extract of bacteriocins of probiotic bacteria isolated from black tiger shrimp before being precipitated in ammonium sulphate [(NH4) 2SO4].

Probiotics	Activities of bacteriocin crude extract (mm ^2^/mL)		
	*V. alginolyticus*	*A. hydrophila*	*P. stutzeri*
H1	(674.65±369.34) ^ab^	(-5.59±530.69) ^b^	(287.078±127.834) ^bc^
H2	(409.38±56.01) ^ab^	(193.31±130.33) ^ab^	(262.073± 79.677) ^bc^
H3	(244.66±175.62) ^b^	(208.44±101.49) ^ab^	(338.348±60.722) ^bc^
H4	(117.00±66.13) ^b^	(319.91±101.03) ^ab^	(178.791±61.842) ^b^
H5	(329.93±98.57) ^ab^	(222.30±115.91) ^ab^	(361.823±33.006) ^a^

Note: H = Bacterial Code. Superscript of the same letters indicates no significant differences in the level of 5%. Values were average of triplicate samples ± standard deviation.

After precipitation with ammonium sulphate [(NH4)2SO4], the activity of each of bacteriocins tends to change. The highest activity was performed by isolate H3 against *V alginolyticus*, that was 896.50 mm ^2^/mL ( Table 2). Meanwhile, the lowest activity was indicated by isolate H1 that was 321.47 mm ^2^/mL. This condition was different from that which was before been precipitated with ammonium sulphate. The highest activity was indicated by isolate H1 against *V alginolyticus* ( Table 1). This concluded that precipitation process resulted in the different effect on bacteriocin produced by each of tested bacteria.

**Table 2.** Activities of bacteriocins-crude extract of probiotic bacteria isolated from black tiger shrimp after precipitation in ammonium sulphate [(NH4) 2SO4].

Probiotics	Bateriocins Activities (mm ^2^/mL)		
	*V. alginolyticus*	*A. hydrophila*	*P. stutzeri*
H1	(321.47±80.75) ^cd^	(321.47±80.75) ^bc^	(516.28±145.10) ^ab^
H2	(854.07±55.25) ^a^	(542.69±26.37) ^abc^	(406.82±149.97) ^ab^
H3	(896.50±375.14) ^a^	(642. 86±279.28) ^abc^	(759.39±304.51) ^a^
H4	(610.28±257.32) ^abc^	(872.93±170.02) ^ab^	(887.10±169.08) ^a^
H5	(702.26±156.45) ^ab^	(669.03±79.56) ^abc^	(586.14±409.24) ^ab^

Note: H = Bacterial Code. Superscript of the same letters indicates no significant differences in the level of 5%. Values were average of triplicate samples ± standard deviation.

The activity of the bacteriocins-crude extract of bacterial isolates from prawns against pathogens *V. alginolyticus, A. hydrophila* and *P. stutzeri* was not significantly different (P>0.05), and the highest activity was indicated by isolate W4 against *A. hydrophila* (746.95 mm ^2^/mL). In comparison to bacteriocin produced by bacteria isolated from black tiger shrimp, of which the highest was indicated by isolate H1 against *A. hydrophila*, that was 674.65 mm ^2^/mL. Therefore, the activity of bacteriocin produced by bacteria from prawns was higher than that of black tiger shrimp.

Values for W4 were not significantly different from isolates W2 and W5 (P>0.05), but it was significantly different from W1 and W3 (P <0.05). The highest activity of the bacteriocins-crude extract against *V. alginolyticus* was shown by isolate W4 (645.76 mm ^2^/mL), which was significantly different (P<0.05) from other four isolates. Meanwhile, the highest activity of bacteriocins-crude extract against *P. stutzeri* was indicated by isolate W2 (412.73 mm ^2^/mL), which was significantly different (P <0.05) from the other four isolates.

Bacteriocinactivities of bacterial isolates from prawns against all pathogens ( *V. alginolyticus, A. hydrophila*, and *P. stutzeri*) were not significantly different (P>0.05). The highest activity was produced by isolate W2 against *P. stutzeri*, and the highest inhibitory activity was 1466.96 mm ^2^/mL which was significantly (P> 0.05) different from other four bacteria isolates ( Table 4). However, the activity of isolate W1 was not significantly different from isolate W5 (P> 0.05) against *P. stutzeri* ( Table. 4). Overall, the activity of bacteriocin precipitated in ammonium sulphate {(NH4)2SO4} was higher than bacteriocin before precipitation in ammonium sulphate.

A quantity of 270 mL of bacteriocin-crude extract was obtained after centrifugation, and then it was precipitated with ammonium sulphate salt [(NH4) _2_SO _4_]. In the deposition process at salt saturation level of 80%, a total of 139.32 g of salt ammonium sulphate [(NH4) 2SO4] was added to the crude extract of bacteriocins. The precipitated bacteriocin was added with buffer in order to reach a volume of 3,375 mL bacteriocins.

**Figure 1.**
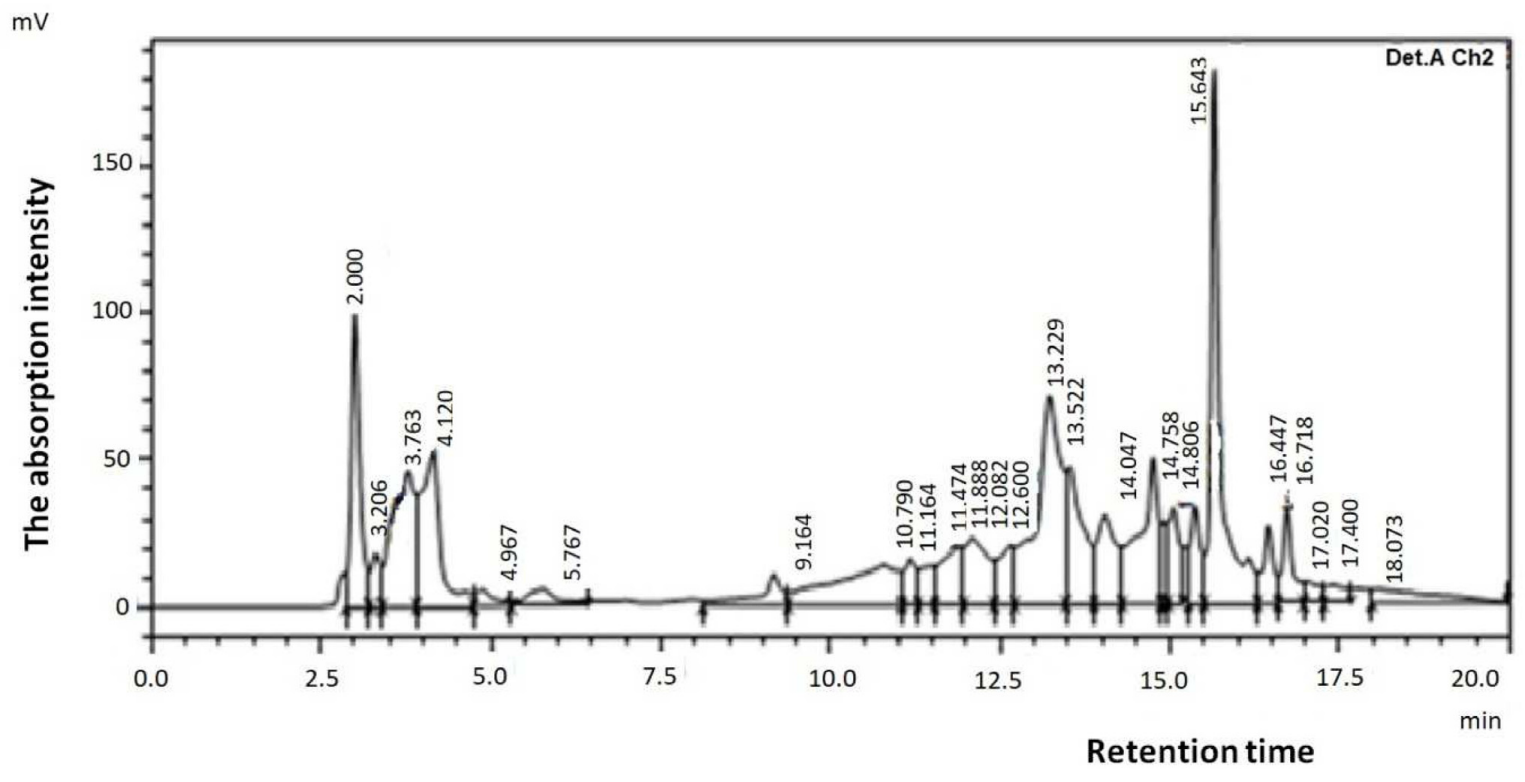
Chromatogram bacteriocins H4 of probiotic bacteria isolated from black tiger shrimp.

**Figure 2.**
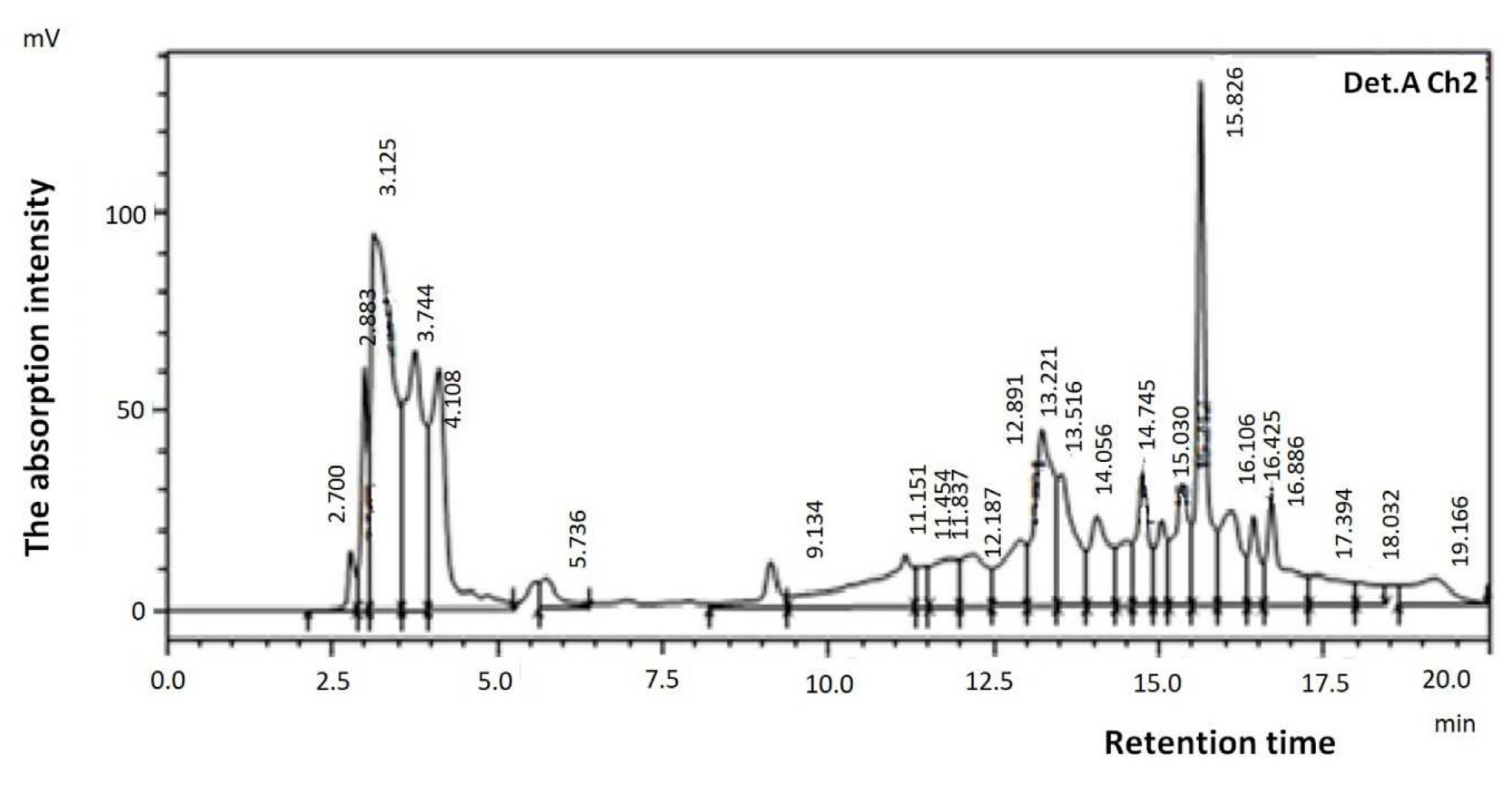
Chromatogram of bacteriocins W2 from probiotic bacteria isolated from prawns.

***HPLC analysis***. High-Performance Liquid Chromatography (HPLC) is a chromatographic method that uses a reversed-phase system as its working system. This method was used to determine the purity level of a compound to be analyzed. Bacteriocins of tiger shrimp and prawns with high bacteriocin-activity values (H4 and W2), were then analyzed by HPLC as shown in Figure 1 and Figure 2. The figures showed that bacteriocin produced by each of probiotic bacteria was not a pure product indicated by the number of chromatogram peaks.

### Gel filtration chromatography

Bacteriocins as a group of proteins, were separated by column chromatography on Sephadex LH-20 using methanol. The resulted eluate was initially appeared light brown in color in the column, and was collected in a vial which was then marked as fraction 1. After that, as the sample was in the middle of the column, the next eluate was collected as fraction 2, and followed by collecting every 20 drop samples until the sample sappeared faded, and finally clear in vial 25. This indicates that the protein samples in the column Sephadex LH-20 have been completely eluted by methanol. The eluate of 25 fractions was allowed to evaporate at room temperature to remove the solvent. The solids obtained were observed to look like thin films in some of the fractions. Five fractions contained thin films of 25 fractions produced, those were fractions 6, 9, 14, 18 and 25, which were then analyzed using FTIR spectroscopy to observe the functional groups containing the fraction.

### FTIR analysis

**Figure 3.**
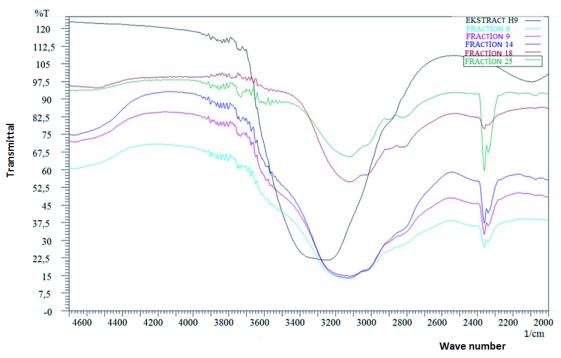
FTIR spectrum bacteriocin H4 precipitated in ammonium sulfate [(NH4) 2SO4] in comparation to the purification product of bacteriocins of some fractions (fraction 6,9,14,18 and 25).

Fourier Transform Infra-Red (FTIR) was used to find out bonding vibration and functional groups contained by bacteriocins. FTIR analysis results from one of the bacteriocins produced by bacteria H4 are shown in Figure 3. The spectrum figure shows the comparison of fractions that produced films (fractions 6, 9, 14, 18 and 25) as the separation products of bacteriocins H4 using gel filtration column, and after being precipitated in ammonium sulphate [(NH4) 2SO4]. Each fraction showed absorption bands at similar wave numbers.

The infrared spectrum of bacteriocins for fraction 6 shows absorption at wave numbers (cm-1), 978, 1340, 1355, 1648, 2831, 2921, 3420 and 3595. Fraction 9 shows absorption at wave numbers (cm-1) of 977, 1089, 1339, 1363, 1404, 1457, 1647, 3239, and 3587. Fraction 14 showed absorption at wave numbers (cm-1) of 978, 1097, 1312, 1363, 1405, 1457, 1647, 2836, and 3502. Fraction 18 shows absorption at wave numbers (cm-1) of 988, 1334, 1358, 1400, 1460, 1649, 2835, 2954, 3415 and 3588. Finally, fraction 25 shows absorption at wave numbers (cm-1) of 971, 1082, 1312, 1339, 1355, 1416, 1457, 1647, 2834, 2988, 3336, 3443 and 3593. Meanwhile, bacteriocins precipitated by salts of ammonium sulphate [(NH4) 2SO4] shows an absorption band at wave number (cm-1) 979, 1339, 1355, 1418, 1456, 3336, 3445, and 3592.

Word document containing the following data tables Crude extract of bacteriocins of probiotic bacteria isolated from black tiger shrimp before being precipitated in ammonium sulphate [(NH4) 2SO4]. The activities of bacteriocins-crude extract of probiotic bacteria isolated from black tiger shrimp after precipitation in ammonium sulphate [(NH4) 2SO4] The activities of bacteriocins-crude extract of probiotic bacteria isolated from prawns before precipitation in ammonium sulphate [(NH4) 2SO4. Click here for additional data file. Copyright: © 2018 Feliatra F et al. 2018 Data associated with the article are available under the terms of the Creative Commons Zero "No rights reserved" data waiver (CC0 1.0 Public domain dedication).

The bacteriocins activities of probiotic bacteria isolated from prawns after precipitation in ammonium sulphate [(NH4)2SO4] Click here for additional data file. Copyright: © 2018 Feliatra F et al. 2018 Data associated with the article are available under the terms of the Creative Commons Zero "No rights reserved" data waiver (CC0 1.0 Public domain dedication).

## Discussion

Bacteriocins are secondary metabolites in the form of proteins that act as antimicrobial compounds. The activity of bacteriocin-crude extracts of bacteria from tiger shrimp before precipitation in ammonium sulphate [(NH _4_) _2_SO _4_] was as presented in Table 1, and after precipitation with the same salt was shown in Table 2. Protein precipitation using 80% ammonium sulphate salt was a partial protein purification technique (partial purification) that is frequently applied as it has high solubility and is easy to obtain. Bacteriocins can be concentrated through the application of methods of salting out using ammonium sulfate as a natural protein ^15^. Salting out is a mechanism of protein precipitation as a result of reduction in solvent molecules required to dissolve the protein. This occurs due to the increase of salt concentration which can decrease protein solubility. When ammonium sulphate is added to the protein solution, the salt ions of ammonium sulphate will attract water molecules away from protein. This is due to the competition between the binding of ions of ammonium sulphate and water which causes the protein precipitates ^16^.

Bacteriocin inhibitory activity against bacterial indicators was indicated as AU (Activity unit). One AU/mL was inhibition area per unit volume of bacteriocin samples tested (mm ^2^/mL). The method used was similar to the antibacterial assay method using agar diffusion method and three Gram-negative bacteria as pathogenic indicators ( *V.alginolyticus, A. hydrophila*, and *P. stutzeri*). 

The bacteriocins-crude extract before being precipitated in ammonium sulphate showed inhibitory activity against three different indicators. The activity of bacteriocin-crude extract of bacterial isolate H4 from tiger shrimp was the highest ( Table 1) against *V. alginolyticus* (117.00 ± 66.13 mm ^2^/mL), *A. hydrophila* (319,91 ± 101.03 mm ^2^/mL and *P. stutzeri* (178.79 ± 61.84 mm ^2^/mL). After precipitation in ammonium sulphate, bacteriocinactivities ( Table 2) increased against *V. alginolyticus* (610.28 ± 257.32 mm ^2^/mL), *A. hydrophila* (872.93 ± 170.02 mm ^2^/mL), and *P. stutzeri* (887.10 ± 169.08 mm ^2^/mL). The H4 isolate showed the greatest inhibitory activity due to its high inhibitory activity against the three bacterial indicators. The better the bacteria inhibit pathogenic bacteria, the better the ability to produce bacteriocins.

Activities of the bacteriocins-crude extract of bacteria from prawns before and after the salt precipitation in ammonium sulphate were were shown in Table 3 and Table 4, respectively. Similar to the inhibitory activity of probiotics bacteria from tiger shrimp, all probiotic bacteria from prawns produced bacteriocins which indicated the active role in inhibiting pathogenic bacteria.

**Table 3.** Activities of bacteriocins-crude extract of probiotic bacteria isolated from prawns before precipitation in ammonium sulphate [(NH4) 2SO4].

Probiotics	Activity of crude extract bacteriocins(mm ^2^/mL)		
	*V. alginolyticus*	*A. hydrophila*	*P. stutzeri*
W1	(310.69±223.32) ^bc^	(238.41±197.97) ^ab^	(314.35±238.86) ^ab^
W2	(345.90±306.16) ^bc^	(455.02±169.59) ^a^	(412.73±209.53) ^a^
W3	(343.00±158.96) ^bc^	(279.41±112.59) ^ab^	(342.36±257.02) ^ab^
W4	(645.76±315.20) ^ab^	(746.95±227.01) ^a^	(92.21±59.37 ) ^ab^
W5	(293.20±197.16) ^bc^	(625.10±296.59) ^a^	(346.00±81.58) ^ab^

Note: W = Bacterial Code. Superscript of the same letters indicates no significant differences in the level of 5%. Values were average of triplicate samples ± standard deviation.

**Table 4.** Bacteriocins activities of probiotic bacteria isolated from prawns after precipitation in ammonium sulphate [(NH4) 2SO4].

Probiotics	Bacteriocins activities(mm ^2^/mL)		
	*V. alginolyticus*	*A. hydrophila*	*P. stutzeri*
W1	(581.03±425.55) ^ab^	(490.88±400.68) ^bc^	(770.87±570.42) ^b^
W2	(1043.2±357,10) ^a^	(1113,91±423.02) ^a^	(1466.96±127.62) ^a^
W3	(607.73±13.94) ^ab^	(531.90±179.18) ^bc^	(461.59±341.75) ^bc^
W4	(367.01±86.39) ^b^	(917.90±186.53) ^ab^	(238.42±35.42) ^c^
W5	(706.18±365.07) ^ab^	(666.49±268.47) ^ab^	(888.76±360.27) ^b^

Note: W Bacterial Code. Superscript of the same letters indicates no significant differences in the level of 5%. Values were average of triplicate samples ± standard deviation.

The average activity of bacteriocins-crude extract from bacteria W2 before salt-precipitation in ammonium sulphate ( Table 4) against *V. alginolyticus*, *A. hydrophila* and *P. stutzeri* were 345.907 ± 306.160 mm ^2^/mL, 455.022 ± 169.591 mm ^2^/mL and 412.735 ± 209.537 mm ^2^/mL, respectively. After the salt-precipitation in ammonium sulfate, the bacteriocin activities increase against *V. alginolyticus*, *A. hydrophila* and *P. stutzeri*, which were 1043.228 ± 357.102 mm ^2^/mL, 1113.914 ± 423.026 mm ^2^/mL and 1466, 127.626 ± 962 mm ^2^/mL, respectively. Activities of bacteriocins produced by bacteria W2 demonstrated the greatest potential to inhibit all three pathogenic indicator bacteria. Ponce *et al*., ^17^ found that bacteriocins of *Lactococcuslactis* have inhibitory activity against *L. monocytogenes* which was 83.33 mm ^2^/mL. This value was lower than the bacteriocin activity of bacteria W2 (1043.228 mm ^2^/mL) against *V. alginolyticus*. This is likely due to the inhibition ability of bacteria W2 being closely related to the bacterial cell growth. The better the cell growth, the number of cells increases, and this will further increase the production of bacteriocins.

Antimicrobial compounds such as bacteriocins are proteins produced as secondary metabolites. The production of secondary metabolites is encoded by DNA-containing genes. Formation of secondary metabolites was affected by several conditions, among which are limitations of available nutrients in the media, the addition of inducer compounds and decrease in the growth rate ^18^.

Comparing the activity of bacteriocins produced by both bacteria (H5 and W2), probiotic bacteria W2 from prawns had the highest activity against the three bacterial indicators. The significant difference (P> 0.05) in the inhibition of the three bacterial indicators showed that the active protein compounds (bacteriocins) were also different ^19^. The sensitivity of bacteria to bacteriocins is determined by the specific characteristics possessed by each bacteria. Thus, inhibition mechanism of bacteriocins against indicator bacteria depends on the specific receptors possessed by the bacteria.

The high inhibitory activity caused by bacterial bacteriocins is closely related to the bacterial indicators which belong to Gram-negative bacteria as shown in Table 5. Bacteriocins produced by Gram-positive bacteria have low inhibitory activity, or none at all, against Gram-negative bacteria ^20^. For example, the activity generated by *Lactobacillus lactis* against *E. coli*is very low ^21^. Bacteriocins can inhibit or kill pathogenic bacteria when the bacteria have a close relationship ^19^. Such as bacteriocins produced by *Lactobacillus* sp. SCG 1223 has a high inhibitory activity against *L. monocytogenes* (1648.500 mm ^2^/mL) ^22^. High activity was also obtained from bacteriocins produced by *Lactobacillus lactis* against *Bacillus subtilis*, *Bacillus megaterium*, *Bacillus cereus*, *Staphylococcus aureus* and *Enterococcus faecalis* ^21^.

**Table 5.** Gram stain test the probiotic bacteria of tiger shrimp and prawns.

Code isolates of tiger shrimp	Gram	Code isolates of tiger shrimp	Gram
H1	(+)	W1	(+)
H2	(-)	W2	(-)
H3	(-)	W3	(-)
H4	(-)	W4	(-)
H5	(+)	W5	(+)

Bacteriocins analysis was performed by HPLC system after purified with salt ammonium sulfate [(NH4) 2SO4]. This analysis used a wavelength of 210 nm and 250 nm because, at these wavelengths, bacteriocins produced by *Lactococcus mesentoroides* show high purity ^13^. The mobile phase used was methanol while the steady phase was silica gel. Bacteriocins analysis results were shown as in Figure 1 and Figure 2. Bacteriocins produced by all the probiotic bacteria after analysis cannot be considered as the pure product because many peaks appear in the chromatogram analysis. This proves that the new protein sample was just partially purified using ammonium sulfate salt [(NH4) 2SO4]. Bacteriocins were protein compounds that the separation should be done with a variety of purification methods as has been done by Smaoui *et al*., ^23^, bacteriocins with code BacTN635 initially purified by ammonium sulfate [(NH4) 2SO4], followed by gel filtration chromatography, HPLC, and SDS-PAGE electrophoresis. Nisin isolated from *Lactococcus lactis* was also purified by various purification methods, which was initially precipitated with ammonium sulphate salt [(NH4)2SO4], and was continued by purification using various chromatographic methods ^8, 24^. Nissin was the first type bacteriocin allowed as biopreservation on food ^5^.

Bacteriocins were produced by probiotic bacteria H4 from tiger shrimp and probiotic bacteria W2 from prawns which were purified by gel filtration chromatography using Sephadex LH-20. The results of the sample separation were characterized by FTIR spectroscopy to identify functional groups contained in the functional group of the bacteriocins based on the literature of various types of bacteriocins by Sakhamuri *et al*., ^24^ and Selvendran and Babu ^25^ as shown in Table 6.

**Table 6.** FTIR characteristic wave numbers of different types of bacteriocins by Sakhamuri *et al*., ^24^, Selvendran and Babu ^25^.

Phase Number	Functional groups
976	OCN
	vibration buckling
1079	P=O
	Fosfodiester
1180-1360	C-N
	peptide bond
1650	C=O
	carbonyl stretching vibration
3336	N-H
3500	OH

The infrared spectrum of bacteriocins H4 at wave numbers (cm-1) of 971, 977, 978, 979, and 988 indicated the presence of bending vibration of amide group (O= oxygen, C= carbon, N= nitrogen), while the wave numbers (cm-1) of 1082, 1089 and 1079 indicated a bond phosphodiester (P = O), and the wave number (cm-1) of 1647, 1648, 1649 and 1652 indicated a stretching vibration of carbonyl (C = O, amide I). The infrared spectrum bacteriocins obtained relate to specific functional groups of nisin which was similar to that reported by Sakhamuri *et al*. ^24^. The wave numbers (cm ^-1^) 3336, 3415, 3420, 3443, and 3445 indicated the presence of N-H stretching vibration. Bacteriocins produced was the result of probiotic bacteria isolated from black tiger shrimp and prawns, one of which has 99% homologous with the bacteria *Bacillus* sp. ^26^.

## Conclusions

Bacteriocins had been explored from probiotic bacteria isolated from black tiger shrimp and prawns. The bacteriocins show potential as anti-pathogens in shrimp culture or in fish farming. Bacteriocins of from H4 isolate from tiger shrimp and bacteriocins of W2 isolate from prawns were two antimicrobial compounds which had the greatest inhibitory activity against all three pathogens, *Vibrio alginolyticus*, *Aeromonas hydrophila* and *Pseudomonas stutzeri*. HPLC analysis of the bacteriocins produced by 18 of probiotic bacteria did not show a high degree of purity. FTIR analysis of the purified products of H4 bacteriocins showed an amide bond at a wavelength of 1652 cm-1 which indicated that the compound was a protein. Bacteriocins produced from the laboratory isolates may be useful as a food preservative for controlling microbial deterioration, enhancing the hygienic quality, and extending the self-life of fish and seafood products ^27^.

## Data availability

The data referenced by this article are under copyright with the following copyright statement: Copyright: © 2018 Feliatra F et al.

Data associated with the article are available under the terms of the Creative Commons Zero "No rights reserved" data waiver (CC0 1.0 Public domain dedication). 

Dataset 1: Word document containing the following data tables: 10.5256/f1000research.13958.d198458 ^28^

Crude extract of bacteriocins of probiotic bacteria isolated from black tiger shrimp before being precipitated in ammonium sulphate [(NH4) 2SO4].

The activities of bacteriocins-crude extract of probiotic bacteria isolated from black tiger shrimp after precipitation in ammonium sulphate [(NH4) 2SO4].

The activities of bacteriocins-crude extract of probiotic bacteria isolated from prawns before precipitation in ammonium sulphate [(NH4) 2SO4.

Dataset 2: The bacteriocins activities of probiotic bacteria isolated from prawns after precipitation in ammonium sulphate [(NH4)2SO4]. 10.5256/f1000research.13958.d198459 ^29^
