# Supplementary material for: Potential of bacteriocins produced by probiotic bacteria isolated from tiger shrimp and prawns as antibacterial to Vibrio, Pseudomonas, and Aeromonas species on fish
Source: F1000Res. 2018 Mar 29;7:415. [Version 1] doi: 10.12688/f1000research.13958.1 (PMC6182674; doi:10.12688/f1000research.13958.1)
Supplement: Word document containing the following data tables — Crude extract of bacteriocins of probiotic bacteria isolated from black tiger shrimp before being precipitated in ammonium sulphate [(NH4) 2SO4]. The activities of bacteriocins-crude extract of probiotic bacteria isolated from black tiger shrimp after precipitation in ammonium sulphate [(NH4) 2SO4] The activities of bacteriocins-crude extract of probiotic bacteria isolated from prawns before precipitation in ammonium sulphate [(NH4) 2SO4. [file f1000research-7-15172-s0000.tgz › 87fd6121-4506-4fa9-8fcd-a9fe98ca7298_Raw_data-tables.docx]

**Raw data:**

**Summary of crude extract of bacteriocins of probiotic bacteria isolated from black tiger shrimp before being precipitated in ammonium sulphate [(NH4) 2SO4]**

| **Probiotic** | **Replica** | **Indicator Bacteria** | | | **Activities of bacteriocin crude extract (mm^2^/mL)** | | | **Average** | | |
| --- | --- | --- | --- | --- | --- | --- | --- | --- | --- | --- |
|  |  | ***Vibrio*** | ***Aeromonas*** | ***Pseudomonas*** | ***Vibrio*** | ***Aeromonas*** | ***Pseudomonas*** | ***Vibrio*** | ***Aeromonas*** | ***Pseudomonas*** |
| H1 | I | 7.5 | 8.2 | 7.66 | 44.1563 | 52.7834 | 46.0603 | 61.9929 | 27.9800 | 42.6139 |
|  | II | 10.16 | 6.3 | 7.7 | 81.0321 | 31.1567 | 46.5427 |  |  |  |
|  | III | 8.8 | 0 | 6.7 | 60.7904 | 0.0000 | 35.2387 |  |  |  |
| H2 | I | 8.13 | 6.3 | 6.9 | 51.8861 | 31.1567 | 37.3739 | 48.7294 | 37.9260 | 41.3636 |
|  | II | 7.8 | 7 | 7.6 | 47.7594 | 38.4650 | 45.3416 |  |  |  |
|  | III | 7.7 | 7.5 | 7.26 | 46.5427 | 44.1563 | 41.3755 |  |  |  |
| H3 | I | 7.23 | 7.53 | 7.3 | 41.0342 | 44.5102 | 41.8327 | 40.4934 | 38.6824 | 45.1774 |
|  | II | 7.9 | 6.7 | 7.8 | 48.9919 | 35.2387 | 47.7594 |  |  |  |
|  | III | 6.33 | 6.8 | 7.65 | 31.4541 | 36.2984 | 45.9402 |  |  |  |
| H4 | I | 6.9 | 7 | 6.78 | 37.3739 | 38.4650 | 36.0852 | 34.1102 | 44.2557 | 37.1996 |
|  | II | 6.26 | 7.8 | 7.2 | 30.7623 | 47.7594 | 40.6944 |  |  |  |
|  | III | 6.6 | 7.7 | 6.66 | 34.1946 | 46.5427 | 34.8191 |  |  |  |
| H5 | I | 7.2 | 7.66 | 7.66 | 40.6944 | 46.0603 | 46.0603 | 44.7567 | 39.3751 | 46.3511 |
|  | II | 8 | 6.8 | 7.83 | 50.2400 | 36.2984 | 48.1275 |  |  |  |
|  | III | 7.43 | 6.75 | 7.56 | 43.3358 | 35.7666 | 44.8656 |  |  |  |

**Activities of bacteriocins-crude extract of probiotic bacteria isolated from black tiger shrimp after precipitation in ammonium sulphate [(NH4) 2SO4]**

| **Probiotic** | **Replica** | **Indicator Bacteria** | | | **Activities of bacteriocin (mm^2^/mL)** | | | **Average** | | |
| --- | --- | --- | --- | --- | --- | --- | --- | --- | --- | --- |
|  |  | ***Vibrio*** | ***Aeromonas*** | ***Pseudomonas*** | ***Vibrio*** | ***Aeromonas*** | ***Pseudomonas*** | ***Vibrio*** | ***Aeromonas*** | ***Pseudomonas*** |
| H1 | I | 7.9 | 7.9 | 8.6 | 48.9919 | 48.9919 | 58.0586 | 44.3339 | 44.3339 | 54.0744 |
|  | II | 7.3 | 7.3 | 8.63 | 41.8327 | 41.8327 | 58.4644 |  |  |  |
|  | III | 7.33 | 7.33 | 7.63 | 42.1772 | 42.1772 | 45.7003 |  |  |  |
| H2 | I | 9.3 | 8.3 | 7.4 | 67.8947 | 54.0787 | 42.9866 | 70.9638 | 55.3948 | 48.6014 |
|  | II | 9.66 | 8.4 | 7.63 | 73.2527 | 55.3896 | 45.7003 |  |  |  |
|  | III | 9.56 | 8.5 | 8.53 | 71.7440 | 56.7163 | 57.1173 |  |  |  |
| H3 | I | 10.9 | 9.8 | 9.3 | 93.2659 | 75.3914 | 67.8947 | 73.0852 | 60.4031 | 66.2296 |
|  | II | 8.46 | 8.6 | 8 | 56.1837 | 58.0586 | 50.2400 |  |  |  |
|  | III | 9.43 | 7.8 | 10.13 | 69.8060 | 47.7594 | 80.5543 |  |  |  |
| H4 | I | 7.56 | 10.2 | 10.03 | 44.8656 | 81.6714 | 78.9717 | 58.7740 | 71.9066 | 72.6155 |
|  | II | 8.83 | 9.3 | 8.96 | 61.2056 | 67.8947 | 63.0211 |  |  |  |
|  | III | 9.46 | 9.18 | 9.83 | 70.2509 | 66.1538 | 75.8537 |  |  |  |
| H5 | I | 9.46 | 9.13 | 8.8 | 70.2509 | 65.4352 | 60.7904 | 63.3734 | 61.7116 | 57.5674 |
|  | II | 9.1 | 8.56 | 9.1 | 65.0059 | 57.5198 | 65.0059 |  |  |  |
|  | III | 8.36 | 8.9 | 7.73 | 54.8633 | 62.1799 | 46.9060 |  |  |  |

**Activities of bacteriocins-crude extract of probiotic bacteria isolated from prawns before precipitation in ammonium sulphate [(NH4) 2SO4]**

| **Probiotic** | **Replica** | **Indicator Bacteria** | | | **Activities of bacteriocin crude extract (mm^2^/mL)** | | | **Average** | | |
| --- | --- | --- | --- | --- | --- | --- | --- | --- | --- | --- |
|  |  | ***Vibrio*** | ***Aeromonas*** | ***Pseudomonas*** | ***Vibrio*** | ***Aeromonas*** | ***Pseudomonas*** | ***Vibrio*** | ***Aeromonas*** | ***Pseudomonas*** |
| W1 | I | 7.2 | 6.76 | 8.53 | 40.6944 | 35.8726 | 57.1173 | 43.7948 | 40.1809 | 43.9776 |
|  | II | 8.46 | 6.5 | 7.23 | 56.1837 | 33.1663 | 41.0342 |  |  |  |
|  | III | 6.63 | 8.1 | 6.56 | 34.5062 | 51.5039 | 33.7814 |  |  |  |
| W2 | I | 7.63 | 8.8 | 8.8 | 45.7003 | 60.7904 | 60.7904 | 45.5554 | 51.0111 | 48.8967 |
|  | II | 6.2 | 7.7 | 7.23 | 30.1754 | 46.5427 | 41.0342 |  |  |  |
|  | III | 8.8 | 7.63 | 7.56 | 60.7904 | 45.7003 | 44.8656 |  |  |  |
| W3 | I | 7.46 | 7.56 | 8.7 | 43.6865 | 44.8656 | 59.4167 | 45.4101 | 42.2308 | 45.3781 |
|  | II | 7 | 7.66 | 6.6 | 38.4650 | 46.0603 | 34.1946 |  |  |  |
|  | III | 8.3 | 6.75 | 7.36 | 54.0787 | 35.7666 | 42.5231 |  |  |  |
| W4 | I | 8.6 | 8.83 | 6.3 | 58.0586 | 61.2056 | 31.1567 | 60.5480 | 65.6076 | 32.8706 |
|  | II | 9.93 | 8.53 | 6.8 | 77.4048 | 57.1173 | 36.2984 |  |  |  |
|  | III | 7.67 | 10 | 6.3 | 46.1807 | 78.5000 | 31.1567 |  |  |  |
| W5 | I | 6.4 | 7.73 | 7.46 | 32.1536 | 46.9060 | 43.6865 | 42.9202 | 59.5152 | 45.5604 |
|  | II | 7.58 | 8.43 | 8 | 45.1033 | 55.7859 | 50.2400 |  |  |  |
|  | III | 8.1 | 9.83 | 7.38 | 51.5039 | 75.8537 | 42.7546 |  |  |  |

**Bacteriocins activities of probiotic bacteria isolated from prawns after precipitation in ammoniumsulphate [(NH4) 2SO4]**

| **Probiotic** | **Replica** | **Indicator Bacteria** | | | **Activities of bacteriocin (mm^2^/mL)** | | | **Average** | | |
| --- | --- | --- | --- | --- | --- | --- | --- | --- | --- | --- |
|  |  | ***Vibrio*** | ***Aeromonas*** | ***Pseudomonas*** | ***Vibrio*** | ***Aeromonas*** | ***Pseudomonas*** | ***Vibrio*** | ***Aeromonas*** | ***Pseudomonas*** |
| W1 | I | 8.06 | 7.1 | 11.03 | 50.9964 | 39.5719 | 95.5038 | 57.3118 | 52.8040 | 66.8037 |
|  | II | 7.13 | 7.4 | 9.2 | 39.9070 | 42.9866 | 66.4424 |  |  |  |
|  | III | 10.16 | 9.83 | 7 | 81.0321 | 75.8537 | 38.4650 |  |  |  |
| W2 | I | 8.73 | 11.75 | 11.06 | 59.8271 | 108.3791 | 96.0240 | 80.4214 | 83.9557 | 101.6081 |
|  | II | 10.7 | 9.56 | 11.3 | 89.8747 | 71.7440 | 100.2367 |  |  |  |
|  | III | 10.8 | 9.56 | 11.76 | 91.5624 | 71.7440 | 108.5636 |  |  |  |
| W3 | I | 8.7 | 7.56 | 9.5 | 59.4167 | 44.8656 | 70.8463 | 58.6465 | 54.8551 | 51.3397 |
|  | II | 8.63 | 8.56 | 7.5 | 58.4644 | 57.5198 | 44.1563 |  |  |  |
|  | III | 8.6 | 8.9 | 7.05 | 58.0586 | 62.1799 | 39.0165 |  |  |  |
| W4 | I | 8 | 10.36 | 7.03 | 50.2400 | 84.2537 | 38.7954 | 46.6107 | 74.1551 | 40.1815 |
|  | II | 7.3 | 9.16 | 7.33 | 41.8327 | 65.8659 | 42.1772 |  |  |  |
|  | III | 7.8 | 9.6 | 7.1 | 47.7594 | 72.3456 | 39.5719 |  |  |  |
| W5 | I | 7.36 | 9.6 | 9.6 | 42.5231 | 72.3456 | 72.3456 | 63.5694 | 61.5847 | 72.6981 |
|  | II | 9.65 | 9.16 | 10.76 | 73.1012 | 65.8659 | 90.8854 |  |  |  |
|  | III | 9.78 | 7.7 | 8.36 | 75.0840 | 46.5427 | 54.8633 |  |  |  |
