# Supplementary material for: Potential of bacteriocins produced by probiotic bacteria isolated from tiger shrimp and prawns as antibacterial to Vibrio, Pseudomonas, and Aeromonas species on fish
Source: F1000Res. 2018 Mar 29;7:415. [Version 1] doi: 10.12688/f1000research.13958.1 (PMC6182674; doi:10.12688/f1000research.13958.1)
Supplement: The bacteriocins activities of probiotic bacteria isolated from prawns after precipitation in ammonium sulphate [(NH4)2SO4] [file f1000research-7-15172-s0001.tgz › bc208f94-88f1-4765-b993-8d456c1e4cab_Raw_data-FTIR_10_-Labelled.docx]

**Wave Lenght (1/cm)**

**Transmittal (%T)**

FTIR spectrum bacteriocin H4 precipitated in ammonium sulfate [(NH4) 2SO4] in comparationto the purification product of bacteriocins of some fractions (fraction 6,9,14,18 and 25)

**Wave Lenght (1/cm)**

**Transmittal (%T)**

FTIR spectrum bacteriocin H4 precipitated in ammonium sulfate [(NH4) 2SO4] in comparationto the purification product of bacteriocins of some fractions (fraction 6,9,14,18 and 25) different peaks

**Wave Lenght (1/cm)**

**Transmittal (%T)**

FTIR spectrum bacteriocin H4 precipitated in am monium sulfate [(NH4) 2SO4] in comparationto the purification product of bacteriocins of some fractions (fraction 6,9,14,18 and 25) different peaks

**Wave Lenght (1/cm)**

**Transmittal (%T)**

FTIR spectrum bacteriocin H4 precipitated in ammonium sulfate [(NH4) 2SO4] in comparationto the purification product of bacteriocins of some fractions (fraction 6,9,14,18 and 25) different peaks

**Wave Lenght (1/cm)**

**Transmittal (%T)**

FTIR spectrum bacteriocin H4 precipitated in ammonium sulfate [(NH4) 2SO4] in comparationto the purification product of bacteriocins of some fractions (fraction 6,9,14,18 and 25) different peaks

figure. 3 FTIR spectrum bacteriocin H4 precipitated in ammonium sulfate [(NH4) 2SO4] in comparationto the purification product of bacteriocins of some fractions (fraction 6,9,14,18 and 25) different peaks
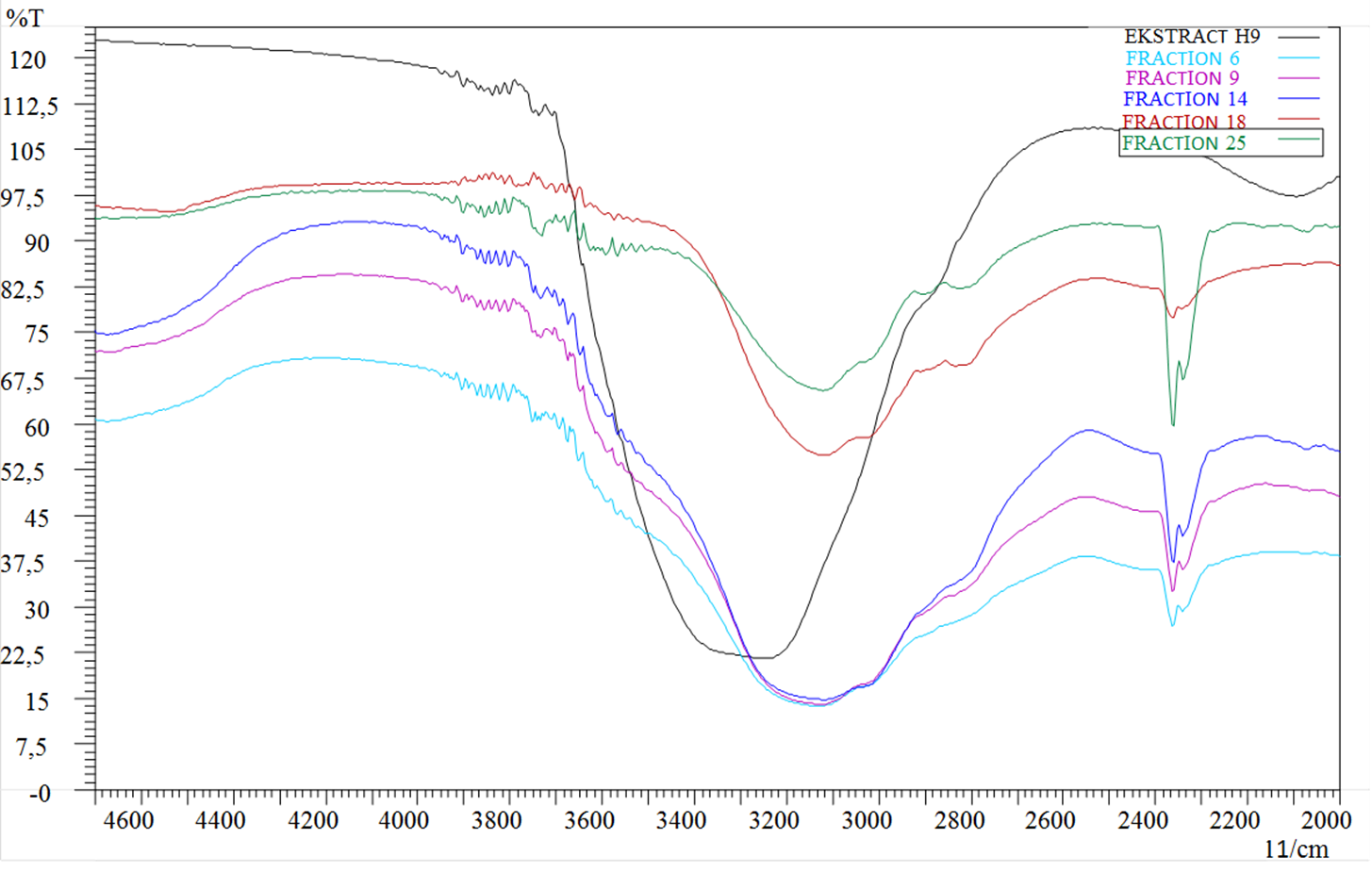


**Transmittal (%T)**

**Wave Lenght (1/cm)**

FTIR spectrum bacteriocin H4 precipitated in ammonium sulfate [(NH4) 2SO4] in comparationto the purification product of bacteriocins of some fractions (fraction 6,9,14,18 and 25) different peaks
