# Supplementary material for: Potential of bacteriocins produced by probiotic bacteria isolated from tiger shrimp and prawns as antibacterial to Vibrio,  Pseudomonas, and Aeromonas species on fish
Source: F1000Res. 2018 Mar 29;7:415. [Version 1] doi: 10.12688/f1000research.13958.1 (PMC6182674; doi:10.12688/f1000research.13958.1)
Supplement: Peer review history

# Peer review: Referee response for version 1

**DOI:** 10.5256/f1000research.15172.r38187

**Published:** 2018-10-10

**Authors:** Eddy Afrianto

**Affiliations:** 1 Faculty of Fisheries and Marine Sciences, Padjadjaran University, Jatinangor, West Java, Indonesia

In the field of fisheries, the attack of pathogenic microbes and the use of formaldehyde are some of the main issues. The use of formalin as a food preservative has reached dangerous stage, but its more effective substitute compounds have not been successfully developed. Bacteriocin is a compound that has great potential to be developed as a prevent attacks of pathogenic bacteria and preservative inhibitor to replace formaldehyde.

 Bacteriocin is a secondary metabolite product from lactic acid bacteria (BAL). These bacteria are often found in various places, including the digestive tract of fish and shrimp.  There have been many efforts made to get bacteriocin-producing microbes.  Increased mass production of bacteriocin is very helpful for fish cultivation activities in preventing pathogenic microbial attack and processing of fishery products as preservatives replacing formalin.

 This research is the initial stage of the effort to develop bacteriocin as a deterrent to the activity of pathogenic microbes and decay. Bacterial isolates that have been found need to go through several stages in order to produce bacteriocins, including isolation processes to produce pure cultures and optimal bacteriocin separation technology from microbial culture media.

1. In the abstract several words are repeated, reducing the use efficiency of the word.
2. The reasons put forward to carry out this research are quite relevant. It is hoped that bacteriocin can be found that can overcome the problem of pathogens that attack fish and shrimp.
3. The method used has been presented in sufficient detail so as to facilitate future replication. Some parts still need to be detailed further, for example need further explanation on how to get bacteriocins using centrifugation, how many bacteriocin samples are used in the HPLC method, what and how many solvents are used?

I have read this submission. I believe that I have an appropriate level of expertise to confirm that it is of an acceptable scientific standard.

# Peer review: Referee response for version 1

**DOI:** 10.5256/f1000research.15172.r38186

**Published:** 2018-09-20

**Authors:** Tati Nurhayati

**Affiliations:** 1 Department of Aquatic Product Technology, Faculty of Fisheries and Marine Science, Bogor Agricultural University, Bogor, Indonesia

The manuscript describes the isolation of bacteriocin derived from probiotic bacteria isolated from shrimp and lobster. This compound is tested against pathogenic bacteria. However, there are some improvement notes in some parts.

 Abstract

1. The research objective in the abstract has not been clearly written. Are bacteriocins obtained from shrimp or from bacteria that live on shrimp?
2. There is a typo in the method section in the abstract. Bacteriiocin was written 2 times.
3. In the abstract: the sentence Bacteriocin activity against is written 2 times.
4. The results of chromatographic column analysis and HPLC have not been written in the results section of the abstract.

Methodology

1. Assay of bacteriocin activity, need to be re-examined. Need to be written clearly, how many bacteriocins are inserted into each well? In order to be able to track how to calculate the inhibition per ml. Unit diameter should be mm NOT mm2.
2. In methodology purification methods, the use of ammonium sulfate, only one concentration is 80%. What is the basis? If there have been previous studies, it is necessary to mention here referring to whose research? Use of the term the purification of proteins with ammonium sulfate is inappropriate.
3. There is a discrepancy in the designation term. In the abstract, it is stated that purification is done by chromatography column. But in the methodology, protein purification is carried out with ammonium sulfate.
4. In the methodology, there is step gel filtration. What is the use of gel filtration for? For purification only, or for specific analysis. Need to write a clear title in this method.
5. In the methodology, analysis with HPLC is used to analyze what. Need to be written in the methodology.

Discussion

1. Especially for the discussion of gel filtration, only the stages of the method are explained. The method stage should be written on the methodology not in the results and discussion.
2. In sub discussion, it is better not to repeat mentioning the actual data already in the results section. In this sub-chapter, what should be written is the discussion of the data in the results section.

Conclusion

1. The last sentence from the conclusion is incorrect, because the researchers did not test bacteriocin activity against spoilage bacteria but against pathogenic bacteria.

I have read this submission. I believe that I have an appropriate level of expertise to confirm that it is of an acceptable scientific standard, however I have significant reservations, as outlined above.

# Peer review: Referee response for version 1

**DOI:** 10.5256/f1000research.15172.r37670

**Published:** 2018-09-07

**Authors:** Sriram Seshadri

**Affiliations:** 1 Institute of Science, Nirma University, Ahmedabad, India

1. Lot of words are repeating in the same sentence in the abstract.
2. How do you produce bacteriocin by centrifugation?
3. The species name of shrimp and prawn is missing in the whole test.
4. How was the isolates obtained? What was the sample used?
5. How was the health status of the shrimp and prawn confirmed?
6. Characterization of probiotic features are completely missing.
7. Have the authors confirmed whether the isolates by itself had antibacterial potentials?
8. If so, have they determined the mode of action before moving on to bacteriocin isolation and purification?
9. How do the authors justify selection of only Amoxicillin as the test antobiotics?
10. There are no citations provided for Gel filtration chomatography.
11. There is no mention of standard probiotic strain which are known to produce bacteriocins. How did the authors compare the bacteriocin production and its efficacy?
12. The chemical formula of Ammonium Sulphate is (NH _4_) _2_SO _4_ not (NH4)2SO4. It has to be rectified throughout the text.
13. Bacteriocin inhibitory activity is indicated as AU. AU does not stand for Activity Unit by Arbitrary Unit.
14. Discussion is very poorly written. It has to be re-written and there is no mention of any papers on bacteriocins isolated from prawn/shrimps/fish sources.

I have read this submission. I believe that I have an appropriate level of expertise to state that I do not consider it to be of an acceptable scientific standard, for reasons outlined above.

## References

[1] SahooTJenaPPatelASeshadriS: Bacteriocins and their applications for the treatment of bacterial diseases in aquaculture: a review. *Aquaculture Research*.2016;47(4) : 10.1111/are.12556 1013-1027 10.1111/are.12556

[2] SahooTKJenaPKPatelAKSeshadriS: Purification and Molecular Characterization of the Novel Highly Potent Bacteriocin TSU4 Produced by Lactobacillus animalis TSU4. *Appl Biochem Biotechnol*.2015;177(1) : 10.1007/s12010-015-1730-z 90-104 10.1007/s12010-015-1730-z 26245257

# Peer review: Referee response for version 1

**DOI:** 10.5256/f1000research.15172.r32653

**Published:** 2018-04-24

**Authors:** Hazel Monica Matias-Perala

**Affiliations:** 1 Department of Technology and Heritage, Faculty of Science, Technology and Human Development, Tun Hussein Onn University of Malaysia (UTHM), Batu Pahat, Malaysia

The manuscript contained important results from a research activity aimed to isolate potential probiotic bacteria which produce potent bacteriocin. Upon isolation of bacteriocins, they were tested for potential antibacterial activity against pathogenic bacteria, through the utilization of disc diffusion method/ assay. After which, the bacteria that perform the best in the disc diffusion method was chosen (one from the tiger shrimp (H) and another one from freshwater prawn (W)) was further purified and subjected to HPLC and FTIR analysis. 

- The Introduction section clearly stated the importance of doing this research which is the utilization of bacteriocin to help in alleviating the problems of pathogens in aquaculture particularly the fish and shrimp culture. The authors also cited recent references to indicate the timeliness of this work.
- The methods presented are clear with enough details for easy replication in the future. However, few details were left out in the HPLC analysis which has to be added to the manuscript: (Please indicate in the HPLC method the information on how much (microliter) of the bacteriocin samples were injected, what is the solvent used, how many percents of solvent are added and etc. 
- The statistical analysis is appropriate. Although it is suggested to add up which dataset were subjected to ANOVA and DMRT.
- There are few things to clear up in the results section. 
  - Please check the results which the author considered "significantly different" throughout the results. Similar letters indicate no significance, therefore as an example, "ab", "bc", and "b" are not significantly different. 
  - Table 1, H1 - under A. hydrophila, indicate the real value (there seemed to be a problem).
  - Table 3 was not cited in the text.
  - Why is Table 5 not in presented in the "Results" section, but is discussed in the "Discussion" section? If the data tabulated in Table 5 is from your own research, it is more appropriate to present in the results section, rather than only cite in the Discussion section.
- The results are considered only partially discussed in the  Discussion section. Therefore it will be best to add more appropriate justifications. Following are suggested in the discussion section for improvement:
  - Tables and figures should only be cited minimally in the discussion section since they have already been presented in the results section.
  - Explain more the implication of increasing inhibitory activity against pathogens after precipitation in ammonium sulfate [(NH4) 2SO4]. Will it help in terms of increased efficiency of bacteriocin? How about in terms of the economics?
  - It will be best to expound on the result of the inhibitory activity of the bacteriocin comparing the current results to that of those previously been used. In the methodology, there was a mention of positive and negative control but the result was not used in the argument on how efficient the currently isolated and purified bacteriocin in this study.
  - There should also be a clear explanation of the reason why H4 and W2 were chosen among other isolates. In here the statistical results will become handy in explaining why.
  - Include more explanation on the FTIR results for the two chosen isolate which are potentially good source of potent bacteriocin. What were the implications of the FTIR results having the specific wave numbers as in the current results? Do they mean more potent? better than others?
- As for the conclusion section: It is not advisable to cite any reference in the conclusion section since conclusion should contain the authors' own interpretation of the results collected from the study.

I have read this submission. I believe that I have an appropriate level of expertise to confirm that it is of an acceptable scientific standard.
